# Cost-Effective Training Models in Point-of-Care Ultrasound for Medical Students in Emergency Medicine: An Evaluation of Current Resources

**DOI:** 10.7759/cureus.23753

**Published:** 2022-04-02

**Authors:** Jordan M Palmer, Andrew Little, Quoc Vinh Tran

**Affiliations:** 1 Education, Alabama College of Osteopathic Medicine, Dothan, USA; 2 Emergency Medicine, AdventHealth East Orlando, Orlando, USA

**Keywords:** point-of-care ultrasound, simulation trainer, medical student education, teaching in emergency medicine, ultrasound education, simulation in medical education, emergency medical procedures

## Abstract

Background

Ultrasound is becoming more widely utilized in clinical practice; however, its effectiveness is limited by the operator’s skills. Simulation models are attractive options for developing skills because they allow inexperienced users to practice without the risk of endangering patients.

Objective

The purpose of this study was to identify commercially available and homemade ultrasound models to describe them in terms of materials, cost, and whether they are high- or low-fidelity for medical student education.

Methods

This is an investigational study on cost-effective ultrasound training methods for medical students. Our study was performed using search engines in Google, Google Scholar, and PubMed to search for models for the following five modalities: foreign body identification, intravenous (IV) injection training, abdominal ultrasound, ocular ultrasound, and ultrasound-guided lumbar puncture training.

Results

Most homemade models for foreign body identification, IV injection training, and ocular ultrasound could be created for less than $20. IV injection training models were the cheapest commercially available models. There are multiple commercially available options for abdominal ultrasound models, but no options were found for homemade construction. The construction cost for lumbar puncture models was larger due to the need to purchase an anatomically accurate set of lumbar vertebrae.

Conclusions

This study provides initial guidance and suggestions for ultrasound training models that are currently available. Ultrasound models that can be cheaply made or purchased increase accessibility for medical students to gain early exposure in a cost-effective and safe manner.

## Introduction

Point-of-care ultrasonography (POCUS) is an ultrasound that is performed at the bedside to address a specific clinical concern. It is commonly used in the emergency medicine setting because it decreases the amount of time to make a diagnosis as it can be performed and interpreted rapidly. POCUS also reduces patient risk when used to guide procedures [1-3].

Considering that a certain level of competence must be obtained before utilizing POCUS within a clinical setting, ultrasound training is more commonly being included in medical school curricula [4]. Medical students who receive this training and exposure to ultrasound techniques are better suited to make accurate diagnoses using this tool. They are more likely to obtain accurate images and interpret them [5].

Ultrasound simulators are tools that students can use to practice both ultrasound-guided procedures and diagnostic ultrasonography. A variety of commercially available simulators can be constructed, with the main differences being similarity to human tissue and cost [6]. Simulators can be classified as either high-fidelity, typically commercially available, or low-fidelity, typically homemade. Commercially available simulators tend to be much more expensive, ranging from hundreds to tens of thousands of US dollars depending on the target modality. The advantage of using high-fidelity simulators is that they more accurately replicate the anatomy and sonographic appearance of the target tissue [7]. The primary benefit of using homemade low-fidelity simulators is that they are much more inexpensive than high-fidelity simulators, but they are less realistic [7,8].

The focus of this article was to identify commercially available and homemade ultrasound simulators for the following modalities: foreign body identification, ultrasound-guided lumbar puncture, intravenous (IV) injection training, abdominal ultrasound, and ocular ultrasound. These modalities were chosen because they are either commonly performed procedures or represent pathologies that are widely observed in the emergency department. They are also essential to be applied in medical school curricula [9].

This article was previously presented as a meeting abstract at the 2021 American College of Osteopathic Emergency Physicians (ACOEP) Annual Scientific Assembly on October 12, 2021.

## Materials and methods

Data collection

This was an investigational study done by conducting searches on Google, Google Scholar, and PubMed for ultrasound-compatible models for the following modalities: foreign body identification, IV injection training, abdominal ultrasound, ocular ultrasound, and ultrasound-guided lumbar puncture. Search terms of simple keywords included “__ Ultrasound Training Model OR __ Ultrasound Training Simulator,” and a term describing each modality was used. Inserting “Foreign Body,” “IV Injection,” “Abdominal,” “Ocular,” and “Lumbar Puncture” as the specific terms for each modality produced the most meaningful search results. Additional searches included secondary references from bibliographies. Data collection was done during June 2021.

Inclusion criteria

The examinations for websites listing commercially available models included the first five pages of search results within Google in order to find the most products currently marketed and widely available for purchase. Examinations for homemade models included results from Google Scholar and PubMed. We set out to find five models for each modality. When more than five models were found, the commercially available models or the homemade models with the lowest cost of materials were included.

Exclusion criteria

Homemade models that required supplies and ingredients not readily available in the United States were excluded. Homemade models that required access to a 3D printer to be constructed were not included in this article due to the additional cost and skills needed to operate a 3D printer. We excluded commercially available virtual reality simulators that did not involve the use of a tangible model as there was a significant price difference between the two types of models, and excluding virtual reality models allowed for fair comparison due to additional material costs of the tangible models. Models that did not list ultrasound compatibility in the product description were also excluded.

Data analysis

Within each modality listed above, the models were described and compared based on materials, cost, availability (homemade versus commercial), and reference articles for homemade model instructions or manufacturing companies for commercial models.

## Results

Foreign body identification

Foreign bodies that become retained within the soft tissue may lead to complications such as localized infection and inflammation. POCUS can be used for both diagnosis and removal of the retained foreign body [10]. Three commercially available foreign body ultrasound models were found that ranged in price from $599 to $3,049. The Blue Phantom Leg Model with Foreign Body Identification Insert for Ultrasound Training (CAE Healthcare, Sarasota, FL, USA) is a higher fidelity model because it is in the shape of a human leg, and it has additional uses because other inserts can be purchased to help with practicing skills such as DVT identification or ultrasound-guided vascular access. The Blue Phantom Foreign Body Identification Ultrasound Training Model (CAE Healthcare, Sarasota, FL, USA) and the SONOtrain Foreign Body Model (American 3B Scientific, Tucker, GA, USA) are both shaped like a block.

Homemade model construction involves suspending a target (i.e., wood, needles, and cotton) in a bulking agent. The bulking agent is the component that makes up the surrounding material of the model. Ideally, it should be similar in sonographic appearance to human tissue [8,11]. Five articles detailing instructions on foreign body identification model creation were categorized based on the bulking agent used, as this was the most costly aspect of construction for this modality (Table 1). The model created by Wichtel et al. [12] was the most expensive, with an average cost of CAD $31.50 (about USD $25.15) per model. They used silicone foreign bodies as the embedded target submerged in ballistic gel. Other models used meat-based bulking agents such as chicken breast and SPAM (a brand of precooked canned meat) [13,14]. An advantage of using the SPAM model is the ease of construction and the little preparation time needed. The foreign body can be inserted directly, and then, the top can be smoothed over with your fingers, so there are no markings to hint to the student where the foreign body is located [13]. The advantage of using chicken breast is the wide availability and presence of anatomic structures such as blood vessels and nerves. The disadvantage of poultry models is their perishability and the risk of infection with handling raw meats. Qurash et al. [15] describe a model made of gelatin, which is very inexpensive, widely available, and less prone to infection than meat-based models.

**Table 1:** Homemade models by modality “Very inexpensive” indicates that the cost of construction was less than $20. “Inexpensive” indicates that the cost of construction was between $20 and $50. “More expensive” indicates that the cost of construction was between $50 and $150.

Modality	Bulking agent	Reference	Cost	Additional materials
Foreign body identification	Meat (chicken or turkey breast)	Sultan et al. [14]	Very inexpensive	Embedded target (pimiento olives)
Foreign body identification	Tofu	Sultan et al. [14]	Very inexpensive	Embedded target (wood and wire)
Foreign body identification	Meat (SPAM)	Nolting et al. [13]	Very inexpensive	Embedded target (wood, needles, or cotton)
Foreign body identification	Gelatin	Qurash et al. [15]	Very inexpensive	Embedded target (corn starch and gelatin)
Foreign body identification	Ballistic gel	Wichtel et al. [12]	Inexpensive	Embedded target (silicone candy mold and silicone)
IV injection	Meat (chicken or turkey breast)	Rippey et al. [16]	Very inexpensive	Vessels (modeling balloon, thin-walled silicon tubing, and latex silicone tubing)
IV injection	Meat (SPAM)	Nolting et al. [13]	Very inexpensive	Vessels (index finger of a latex glove)
IV injection	Tofu	Johnson [17]	Very inexpensive	Vessels (modeling balloon)
IV injection	Gelatin	Kocharyan et al. [18]	Very inexpensive	Vessels (Penrose drain, ¾ and ¼ inch)
IV injection	Ballistic gel	Morrow et al. [19]	Inexpensive	Vessels (8-mm internal diameter latex tubing)
Ocular ultrasound	Gelatin	Murphy et al. [20]	Very inexpensive	Globe (ping pong ball), optic sheath (clear vinyl tubing)
Ocular ultrasound	Gelatin	Hajat et al. [21]	Very inexpensive	Globe (ping pong ball), optic sheath (3-mm pediatric microcuffed endotracheal tube)
Ocular ultrasound	Gelatin	Jafri et al. [22]	Very inexpensive	Globe (vending machine capsule), optic sheath (ECG lead)
Ocular ultrasound	Gelatin	Cuévas Gonzales [23]	Very inexpensive	Globe (1-mm-thick aluminum rod)
Lumbar puncture	Gelatin	Bellingham et al. [24]	More expensive	Spine model
Lumbar puncture	Ballistic gel	Morrow et al. [19]	Inexpensive	Spine model

IV injection training

Three commercially available ultrasound-compatible IV injection training models were found that ranged in price from $299.98 to $577. The SONOtrain Ultrasound Vein Model (American 3B Scientific, Tucker, GA, USA) was the most expensive. It has three vessels to practice on with different diameters and depths from the surface and adjustable fluid flow. The Blue Phantom™ Branched 4 Vessel Ultrasound Training Block Model (CAE Healthcare, Sarasota, FL, USA) was the second most expensive model. It has the added features of different arrangements of vessels (overlapping branched vessels) for more advanced training. The VH300 3 Vessel Ultrasound Phantom Trainer (Humimic Medical, Greenville, SC, USA) features a curved vessel with a bifurcating branch. The three vessels have different diameters. All three commercially available models are in the shapes of blocks with simulated vessels located within the blocks and are therefore similar in terms of fidelity.

The construction of homemade IV injection training models uses methods that are similar to foreign body identification models. They can be made from the same bulking materials and use similar methods for construction. Still, the main difference is that for IV models, a tubing filled with fluid is typically placed in the bulking material to simulate vessels instead of a foreign body. Most materials used to create simulated vessels are relatively inexpensive. The main difference in price for homemade model construction is due to the use of a more expensive bulking material such as a ballistic gel. Rippey et al. [16] and Johnson [17] describe using modeling balloons as vessels, which are very inexpensive (about $10 for 100 packs) and are widely available. Nolting et al. [13] describe a method that uses a straw to create a tunnel in a block of SPAM (the bulking material), which is then sealed with a latex glove and filled with ultrasound jelly to simulate a vessel. Other simulated vessels can be created from Penrose drain and latex tubing [18,19].

Abdominal ultrasound

Multiple commercially available models for simulation of emergent POCUS procedures such as FAST scans were found. These models were high-fidelity and all much more expensive than the commercially available models for other modalities within this article, with a price range from $3,999 to $28,000. The Blue Phantom™ FAST Exam Real-Time Ultrasound Training Model (CAE Healthcare, Sarasota, FL, USA) was the most expensive model ($28,000) and provide features such as the ability to also practice transthoracic echocardiography (TTE) and pericardiocentesis procedure training. The SonoSkin® Ultrasound Diagnostic Wearable for FAST and eFAST training (Simulab Corporation, Seattle, WA, USA) is the cheapest model ($3,999), includes a wearable trainer placed on a standardized device patient or a mannequin, and includes the software that can display different pathological conditions. No articles describing the construction of homemade abdominal ultrasound models were found.

Ocular ultrasound

No commercially available ocular ultrasound models were found. Four articles discussing the formulation of a homemade ocular ultrasound model were evaluated. Murphy et al. [20] and Hajat et al. [21] used ping pong balls to form the globe, Jafri et al. [22] used a vending machine capsule as the mold for the globe, and Cuévas Gonzales [23] used an aluminum rod to shape the mold of the globe. Jafri et al. [22] described the use of ECG leads, glue, and foreign bodies to simulate different pathologic eye conditions. Hajat et al. [21] used additional material such as a pediatric microcuffed endotracheal tube, which allowed for the simulated pathologic changing of optic nerve sheath diameter to represent increased intracranial pressure and increased fidelity of the model.

Lumbar puncture training

The commercially available models ranged in price from $1,520 to $3,999. Out of the five commercially available models found and listed (Table 2), Simulab Lumbar Puncture/Epidural and Blue Phantom LP and Spinal Epidural had been validated in terms of realism and compatibilities in a study by Vaughan et al. [25]. Two sources were found that described how to make homemade lumbar spine models to practice ultrasound-guided lumbar punctures. Gelatin or ballistic gel were used as the main bulking ingredients, and the cost of construction ranged from ~$30 to $130. All homemade models required the purchase of a lumbar spine model that was the primary determinant of the model expense and varied in price with the cheapest model mentioned by Morrow et al. [19] costing $19.99. The use of ballistic gel as opposed to gelatin increased the price of the homemade model with the ballistic gel being $36.66/4.5 lb (Clear Ballistics, LLC, Fort Smith, AR, USA). The model described by Morrow et al. [19] was less expensive despite using a more expensive bulking agent due to the listed cost of the lumbar spine model, compared to Bellingham et al. [24] who listed the price of the lumbar spine model as CAD $70 (~USD $56).

**Table 2:** Commercially available models by modality

Modality	Product name	Company	Website	Cost
Foreign body identification	Blue Phantom™ Foreign Body Identification Ultrasound Training Model	CAE Healthcare	https://www.bluephantom.com/product/Foreign-Body-Identification-Ultrasound-Training-Model.aspx?cid=524	$599
Foreign body identification	SONOtrain™ Foreign Body Model	American 3B Scientific	https://www.a3bs.com/sonotrain-foreign-body-model-1019636-p121-3b-scientific,p_1397_27466.html	$655
Foreign body identification	Blue Phantom™ Leg with Foreign Body Identification Insert	CAE Healthcare	https://www.bluephantom.com/product/Leg-with-Foreign-Body-Identification-Insert.aspx?cid=408	$3,049
IV injection	VH300 3 Vessel Ultrasound Phantom Trainer	Humimic Medical	https://humimic.com/product/vh300-3-vessel-ultrasound-phantom-trainer-2/	$299.98
IV injection	Blue Phantom™ Branched 4 Vessel Ultrasound Training Block Model	CAE Healthcare	https://www.bluephantom.com/product/Branched-4-Vessel-Ultrasound-Training-Block-Model.aspx?cid=525	$549
IV injection	SONOtrain™ Ultrasound Vein Model	American 3B Scientific	https://www.a3bs.com/sonotrain-ultrasound-vein-model-1019637-p120-3b-scientific,p_1397_27465.html	$577
Abdominal ultrasound	SonoSkin® Ultrasound Diagnostic Wearable For FAST And EFAST Training Package	Simulab Corporation	https://simulab.com/products/sonoskin-c2-ae-ultrasound-diagnostic-wearable-fast-and-efast-training-package	$3,999
Abdominal ultrasound	SonoMan® Diagnostic Ultrasound Simulator	Simulab Corporation	https://www.simulab.com/products/sonoman%C2%AE-diagnostic-ultrasound-simulator https://www.simulab.com/products/fast-module-sonoman%C2%AE-system	$7,919
Abdominal ultrasound	Adult Human Torso – ultrasound training (transparent)	True Phantom Solutions	https://truephantom.com/product/adult-human-torso-sonography-training-transparent/	$14,900
Abdominal ultrasound	Fast-Acute Abdomen Phantom, FAST-ER FAN, with Sonography for Trauma	Kyoto Kagaku Co. Ltd.	https://www.gtsimulators.com/products/fast-acute-abdomen-phantom-fast-er-fan-with-sonography-for-trauma-kkus-5	$16,999
Abdominal ultrasound	Blue Phantom™ FAST Exam Real Time Ultrasound Training Model	CAE Healthcare	https://www.bluephantom.com/product/FAST-Exam-Real-Time-Ultrasound-Training-Model.aspx?cid=455	$28,000
Lumbar puncture	Ultrasound Compatible Lumbar Puncture/ Epidural Simulator	Kyoto Kagaku Co. Ltd.	https://www.kyotokagaku.com/en/products_data/m43e/	$1,520
Lumbar puncture	Lumbar Puncture Simulator	Simulab Corporation	https://www.simulab.com/products/lumbar-puncture-trainer	$1,975
Lumbar puncture	Ultrasound Epidural & Lumbar Puncture Model	Limbs and Things	https://limbsandthings.com/us/products/61002/61002-ultrasound-epidural-lumbar-puncture-model	$2,060
Lumbar puncture	Adult Lumbar Puncture Ultrasound Training Model	Anatomy Lab	https://anatomywarehouse.com/the-anatomy-lab-adult-lumbar-puncture-ultrasound-training-model-a-108519	$2,174
Lumbar puncture	Blue Phantom™ Lumbar Puncture and Spinal Epidural	CAE Healthcare	https://www.bluephantom.com/product/Lumbar-Puncture-and-Spinal-Epidural.aspx	$3,999

## Discussion

Multiple recipes for homemade models are available to construct foreign body identification models and IV injection training models. This is likely due to the simplicity of construction. Methods that involved using meat models such as chicken breast and SPAM forego steps to prepare the bulking agent, and the foreign body or simulated vessels can be placed in the desired position. Meat models are similar in echogenicity to human tissue, but the downside of using meat models is that they are perishable and limited in reusability [6]. Most materials used as foreign bodies or simulated vessels are relatively inexpensive, and the main difference in price for homemade model construction is due to the use of more expensive bulking materials such as a ballistic gel. The price for constructing ultrasound-compatible lumbar puncture training models is higher than the cost of construction for other modalities due to the need to purchase an anatomically accurate set of lumbar vertebrae. This extra cost, however, is minimal compared to commercially available lumbar puncture models.

High-fidelity models should be used for practicing FAST ultrasounds with simulated pathological conditions due to the complication of creating homemade models that accurately mimic the anatomy in this area. Alternatively, live human volunteers can be used to practice image optimization of structures that need to be visualized in a FAST scan, but students will rarely see pathological findings [4].

This study represents a first attempt to compile a suggested list of both commercially available and homemade ultrasound training models for a range of common modalities that are specific to the field of emergency medicine. This information would be valuable for both medical educators and learners as they try to explore the potential options for the most effective ultrasound training. We believe that this study promotes further investigation into the availability of newer and more affordable ultrasound models in medical training.

Limitations

A limitation of this article is that new homemade and commercial models could be available by the time this article is published. The prices of the listed models could also be different since we last searched. Information about commercially available products retrieved from Google was collected from the descriptions listed by the companies selling the products and was not peer-reviewed. There is also a lack of studies directly comparing differences in cost and materials among these five modalities. There is a need for further prospective comparison studies to determine differences in types of models compared to cost. Despite these limitations, our article provides some useful resources and ideas for ultrasound students and educators.

## Conclusions

This study aimed to provide initial guidance on ultrasound training models that are currently available and categorize them based on cost and materials through the search parameters described above. This study cannot make conclusions about learner utilization for each model or the effectiveness in terms of ultrasound education. Medical students and other learners interested in practicing ultrasound-guided techniques will ideally find this article helpful in identifying models to use based on cost and availability.
